# Low-Calorie Cookies Enhanced with Fish Oil-Based Nano-ingredients for Health-Conscious Consumers

**DOI:** 10.1021/acsomega.4c06050

**Published:** 2024-09-06

**Authors:** Raciye Meral, Erol Kına, Zafer Ceylan

**Affiliations:** † Faculty of Engineering, Department of Food Engineering, Van Yüzüncü Yıl University, Van 65080, Turkiye; ‡ Department of Computer Technology, Computer Technology Programme, Van Yüzüncü Yıl University Özalp Vocational School, Van 65100, Turkiye; § Innovan Entrepreneurship Centre, Van 65080, Turkiye; ∥ Faculty of Science, Department, of Molecular Biology and Genetics, Bartin University, Bartin 74000, Turkiye

## Abstract

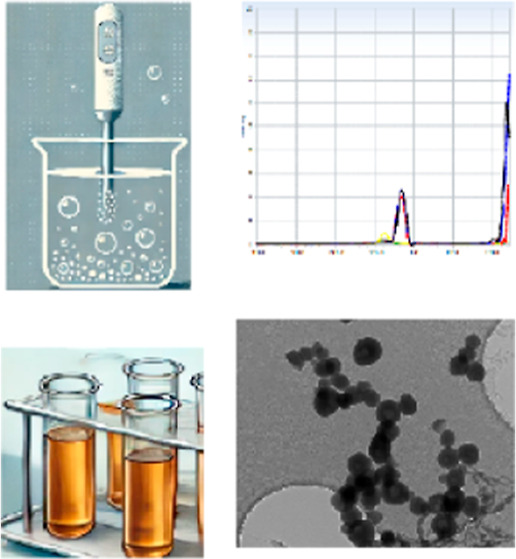

This study explored the effectiveness of fish oil (FO)-loaded nanoemulsions, averaging 197 nm in diameter, as fat substitutes in creating low-calorie cookies. The cookies’ diameter, thickness, and spread ratio were measured, ranging from 46.33 to 57.15 mm, 6.45 to 7.51 mm, and 6.16 to 8.86, respectively. Notably, cookies containing nanoemulsions exhibited a significant increase in the spread ratio compared to the control. The control sample had the highest hardness value at 43.81 N, while the nanoemulsion group had the lowest at 26.98 N. The energy value, which was 508 kcal/100 g in the control group, decreased to 442 kcal/100 g in the group containing the nanoemulsion. The total n-3 fatty acid content in cookies rose from 0.46% in the control cookies to 3.90% in the cookies containing nanoemulsion. Sensory evaluations showed that cookies containing fish ol-loaded nanoemulsion received the highest scores, indicating that the fat reduction did not compromise the desired ″greasy″ sensation. This is especially noteworthy, as it showed that the fat content could be reduced by half without compromising the sensory quality. Utilizing FO-loaded nanoemulsions as a fat replacement in fat-reduced baked goods could provide valuable insights for other food products. The findings have significant implications for the food industry, suggesting that healthier, low-calorie baked goods can be developed without sacrificing physical quality and texture. This approach can cater to the growing market demand for health-conscious food options, potentially leading to new product innovations and enhanced nutritional profiles in a variety of food products.

## 1 Introduction

Shortening, a type of edible fat, is a crucial ingredient in various baked goods, such as pastries, cakes, and cookies. Cookies are among the most beloved bakery products, cherished for their unique flavors and texture. Their defining feature is a low moisture content coupled with high cholesterol and sugar levels. Fat, the second most abundant ingredient in cookie formulations after flour, has a significant impact on the cookie’s structure and flavor. For cookie dough, the fats used must remain solid or semisolid at room temperature, which necessitates a high content of saturated fatty acids (SFA). Shortening plays a vital role in creating the tender texture and pleasant mouthfeel of the final product by preventing the gluten strands from becoming cohesive during mixing.^1−4^ However, recent years have seen a growing health consciousness and a shift toward healthier food choices, necessitating a reduction in the consumption of high-sugar and high-fat products. Excessive fat intake is linked to various health issues, such as obesity, high cholesterol, and coronary heart diseases. Nowadays, consumers are increasingly opting for low-calorie, high-fiber, low-sugar, and low-salt foods to lead a healthier lifestyle. Many researchers^1,2,5,6^ have developed fat-reduced cookies using fat replacers and have examined their physical and sensory quality properties. A significant portion of these studies has reported that the quality parameters, including spread ratio, crunchiness, hardness, and sensory attributes, tend to deteriorate with fat reduction.^7−9^ To address the need for healthier cookie formulations, it is essential to design novel fat replacers that can replicate the flavor, function, and sensory qualities of fat.

The food and agriculture industry has recently turned its attention to nanoemulsion-based delivery systems for proteins, functional foods, and minerals, aiming to enhance productivity and sustain plant and animal tissues.^10^ Nanotechnology presents a promising solution for reducing fat in products like cookies. Nanomaterials offer several advantages over their bulk counterparts, including a greater surface area, the ability to be used in smaller amounts, and higher efficiency.^11^ The increased surface area of nanomaterials allows them to effectively coat the surfaces of dough, thereby enhancing their functional properties in food applications.^12−16^ According to a previous study conducted by Ekin et al. (2019),^12^ nanoformulations of grape seed, sesame oil, black cumin, and coconut oil have been shown to effectively replace 50% of the shortening in cookies without compromising sensory approval from a panel. These findings highlighted the potential of using nanoformulated oils to reduce fat content in baked goods while maintaining their desirable taste and texture characteristics. The use of these nanoemulsions helped in retaining the essential qualities of the cookies, such as flavor and mouthfeel, which are critical for consumer acceptance.^12^ It has been revealed that nanoemulsions prepared with lemon, cinnamon, and citrus oils can be incorporated into cake recipes to enhance their functionality, oxidative stability, and physical qualities without compromising the overall quality. These nanoemulsions not only improve the nutritional profile and shelf life of the cakes but also maintain their desired sensory attributes, making them an excellent alternative for traditional fat and flavor enhancers in bakery products.^17^ Nanoemulsions play a crucial role in nanoencapsulating lipophilic functional foods, as well as in food packaging, edible coatings, and as food ingredients and additives.^18^

Fish oil (FO) consumption is associated with numerous health benefits, including a reduced risk of cardiovascular diseases, various cancers (such as colon, breast, and prostate), and Alzheimer’s disease. FOs are rich in long-chain polyunsaturated omega-3 fatty acids, particularly docosahexaenoic acid (DHA; 22:6n-3) and eicosapentaenoic acid (EPA; 20:5n-3), which are known for their significant health-promoting properties. In the present study, FO-loaded nanoemulsions were evaluated as a potential fat replacer in cookies.

The use of FO-loaded nanoemulsions in cookie formulations aims to leverage these health benefits while maintaining the desirable qualities of the baked products.^19^ Oily fish and FO supplements are the primary dietary sources of these beneficial fatty acids. However, the average intake of long-chain omega-3 polyunsaturated fatty acids remains significantly lower than the recommended minimum of 0.2 g of EPA plus DHA per day. This shortfall is primarily due to the unpleasant taste and odor associated with FO and the general reluctance of people to consume these sources regularly. The development of FO-loaded nanoemulsions as fat replacers in cookies offers a promising solution to enhance omega-3 intake without compromising taste and consumer acceptance.^20^

One way to increase the consumption of FO is to incorporate it into various food formulations. Due to its unique health benefits, particularly the significant levels of unsaturated fatty acids such as DHA and EPA, FO has been widely used in fortified food items. Several foods on the market today have been enhanced with FO, leveraging its nutritional benefits and aiming to overcome the challenges associated with its taste and odor. By adding FO to commonly consumed foods, it becomes easier for people to meet the recommended intake levels of these essential fatty acids, thereby promoting better overall health.^19^

The primary aim of this study was to replace traditional fat with FO-loaded nanoemulsions in cookies without compromising their physical and sensory qualities. A secondary goal was to enrich the cookies with polyunsaturated fatty acids such as EPA and DHA, which are typically absent in conventional cookie formulations. By incorporation of FO-loaded nanoemulsions, the study aimed to develop a fat-reduced product that also offers significant health benefits through increased levels of these essential fatty acids.

## 2 Materials and Methods

### 2.1 Materials

Atlantic mackerel (*Scomber scombrus*) used in this study were purchased fresh from a local seafood market in Van-Türkiye. The mackerel fillets, in glaze form, had an average weight of approximately 150 g and dimensions of 22 cm in length and 6 cm in width. Upon purchase, the fish were immediately transported to the laboratory on ice and processed on the same day to ensure freshness. The Meram Flour Factory provided the flour (Konya, Türkiye). The Sigma-Aldrich Company provided the following ingredients: maltodextrin and Tween 20 (St. Louis, MO, USA).

### 2.2 Extraction of Oil

Fat was extracted from cookies using a cold extraction method, as described by^21^ Bakkalbaşı, Menteş-Yılmaz, Javidipour, and Artık (2012), with some modifications. The samples were ground using a domestic mixer (Bluehouse, Turkey). The cookies were mixed with a solvent (10 times the hexane volume relative to the oil content of the fish fillet) and homogenized using a homogenizer (Heidolph, Germany) at 12,500 rpm for 90 s. The mixture was then filtered, and the extraction process was repeated twice with the residue and fresh hexane (10 times the sample volume). The solvent layer from the pooled filtrates was removed using a rotary vacuum evaporator (Heidolph, Germany).

### 2.3 Fabrication of Nanoemulsion

Nanoemulsion of FO was prepared by first dissolving 1 g of maltodextrin in 9 mL of distilled water using a magnetic stirrer. Then, 1 g of FO and 0.1 g of Tween 20 were added to the solution. The mixture was stirred at 800 rpm with the magnetic stirrer for 30 min to ensure proper dispersion of the oil phase within the aqueous phase, resulting in a coarse emulsion. This coarse emulsion was then subjected to further homogenization using a high-speed homogenizer (Ultra-Turrax T25, IKA) at 12,000 rpm for 5 min, followed by ultrasonication (Bandelin Sonoplus, Heinrichstrabe 3-4d-12207, UW2200, Germany) at 20 kHz for 10 min to achieve a fine nanoemulsion. To determine the optimal percentages of maltodextrin and FO, we conducted preliminary tests. The stability of the emulsions was a crucial factor in these tests. We identified the formulation that remained stable without degradation for 1 month at both 4 and 25 °C.

### 2.4 Particle Size and Zeta Potential

For the dynamic light scattering (DLS), each sample was prepared by diluting the nanoemulsion 1:100 (v/v) in deionized water to avoid multiple scattering effects. The diluted samples were then filtered through a 0.45 μm syringe filter (Millex-HV Filter PVDF; Millipore) to remove dust or large particles before measurement. The DLS technique (using a Zetasizer: Nano ZS, Malvern Instruments Ltd., Worcestershire, UK) was used to measure the emulsion droplet diameter, polydispersity index (PDI), and zeta potential.

### 2.5 Cryogenic Transmission Electron Microscopy

Samples for cryogenic transmission electron microscopy (Cryo-TEM) were prepared by placing a small drop of the nanoemulsion on a carbon-coated copper grid. The excess liquid was blotted with filter paper to form a thin film, which was then rapidly plunged into ethane liquid to vitrify the sample. The vitrified samples were stored in liquid nitrogen until imaging. Cryo-TEM was used to examine the morphology of the created nanoemulsion droplets in their natural condition. A computer-controlled high-resolution electron microscope (Hitachi HT7800, Japan) that runs at 100 kV acceleration voltages was used for the operation. The images were captured at a magnification range of 30,000x–80,000x to visualize the nanoemulsion droplets in detail.

### 2.6 Cookie Preparation

In this study, three different types of cookies were produced: (1) full-fat-containing cookies (control), (2) cookies with a 50% reduction in fat content that contain FO-loaded nanoemulsion (FoN), and (3) cookies with a 50% reduction in fat content without the nanoemulsion (FfC). The water level was reduced taking into account the water level of the nanoemulsion. Cookie formulations are given in Table 1.

**Table 1:** Cookie Formulation^a^

	cookies		
ingredients	Cont	FfC	FoN
wheat flour (g)	100	100	100
sugar (g)	40	40	40
shortening (g)	40	20	20
nanoemulsion (g)	0	0	20
water (g)	22	22	2
sodium bicarbonate (g)	0.75	0.75	0.75
ammonium bicarbonate (g)	0.25	0.25	0.25
corn syrup (g)	1.5	1.5	1.5
salt (g)	1	1	1
vanilla (g)	1	1	1

a Cont: control cookies, FfC: fat reduced and without containing nanoemulsion cookies, and FoN: cookies containing FO-containing nanoemulsion.

Vanilla, salt, sugar, shortening, and sodium bicarbonate were combined and mixed for 3 min at 60 rpm. Water, ammonium bicarbonate, and high-fructose corn syrup were then added to the mixture, and the mixture was thoroughly stirred for 1 min (90 rpm). The dough preparation process was finished after wheat flour was recently added to the formula, and the mixture was mixed for 2 min at 60 rpm. The cookie dough was formed using the techniques suggested by AACC (10–50.05).^22^ The shaped dough was placed on a baking tray and baked in a laboratory oven (Öztiryakiler, İstanbul-Türkiye) at 185 °C for 12 min. The cookies were cooled to room temperature, and then sensory analyses were done. Some of the cooked samples were put in polyethylene bags, sealed, stored at room temperature for 90 days, and used to determine the hardness.

### 2.7 Proximate Composition

According to AOAC (1995)^23^ protocols, the approximate composition of cookies was established.

### 2.8 Evaluation of the Cookie

The diameter of each cookie was measured using a scale, and six cookies were arranged edge to edge. The diameter of six cookies was again measured after each cookie had been rotated 90°, and the average cookie diameter (*D*) was then calculated. Six cookies were placed on top of one another, and their thickness was measured. Following the second measurement of cookie thickness, the average value of the cookie thickness (*T*) was calculated. The spread ratio was calculated from the formula: (*D*/*T*).

### 2.9 Textural Properties of Cookies

Using a texture analyzer and a 3-point bending test with a trigger force of 25 g and a load cell of 50 kg, the hardness of cookies (measured as fracture force) was assessed (model TA-XT2i, Stable Microsystems, U.K.). For the textural investigations, the pre-test speed was 1.5 mm/s, the test speed was 2.0 mm/s, the post-test speed was 10 mm/s, and the distance was 10 mm. The hardness of cookies was assessed 45 and 90 days after they were stored.

### 2.10 Color

A colorimeter (HunterLab, Reston, VA, USA) was used to measure the color of at least four cookies from each batch. The colorimeter was calibrated using white and black standard plates prior to each set of measurements to ensure accuracy. The color measurements were recorded in the CIELAB color space, providing values for L* (lightness), a* (red-green), and b* (yellow-blue). Each cookie was placed flat on the measurement area of the colorimeter, and three readings were taken at different points on the surface of each cookie to account for any potential color variation across the surface. The average of these readings was then calculated to obtain the final L*, a*, and b* values for each cookie. These values were used to analyze and compare the color characteristics of cookies from different batches.

### 2.11 Sensory Evaluation

The sensory panel comprised 30 untrained panelists, ranging in age from 25 to 62 years (17 women and 13 men). While the panelists were not professionally trained, they were familiar with sensory evaluation methods and participated voluntarily. A five-point hedonic scale ranging from 1 (dislike very much) to 5 (like very much) was used by the panelists to rate the acceptability of the cookies. The buying intentions were rated on a scale from 1 (definitely not buy) to 5 (definitely buy).

### 2.12 Determination of Fatty Acids

For the preparation of fatty acid methyl esters (FAMEs), 0.4 g of oil was dissolved in 4 mL of isooctane and methylated with 0.2 mL of 2 M methanolic KOH. The analysis of FAMEs was conducted using an Agilent 6890 N series gas chromatograph equipped with a flame ionization detector and a 30 m fused silica capillary column (INNOWAX, 30 m × 0.25 mm × 0.25 μm; J & W Scientific, USA). The helium carrier gas flow rate was 1.5 mL/min with a split ratio of 100:1. The injector temperature was set at 250 °C, the detector temperature at 260 °C, and the oven temperature was initially set at 120 °C for 5 min, then increased at a rate of 15 °C per minute to 240 °C and held at 240 °C for 20 min. FAMEs were identified by comparing their retention times and equivalent chain lengths with those of standard FAMEs (Supelco 47885-U, Supelco Park, Bellefonte). The sample FAMEs were quantified based on their percentage area.^23^

The following formulas, which were described by Santos-Silva et al.,^24^ Rodríguez et al.,^25^ and Garaffo et al.,^26^ were used to determine the hypocholesterolemic/hypercholesterolemic index (HH), atherogenic index (AI), and thrombogenicity index (TI). The calculations were for the HH, AI, and TI.
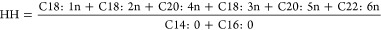

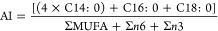

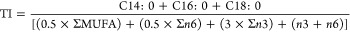


### 2.13 Statistic Evaluation

To identify differences among samples, one-way analysis of variance (ANOVA) was conducted using Python. This analysis was followed by Tukey’s HSD, at a significance level of *p* < 0.05 to determine which samples significantly differed from each other. The statsmodels library in Python was used to perform a one-way ANOVA. After determining that significant differences exist among the groups through ANOVA, Tukey’s HSD test was applied using the SciPy library at a significance level of *p* < 0.05.

## 3 Results and Discussion

### 3.1 Characterization of Nanoemulsion

DLS was used to measure the size distribution of the nanoemulsions. The mean diameters of the droplets and the PDI were found to be 197 nm and 0.224, respectively. The PDI is a crucial parameter in nanoparticle characterization because it provides insights into the uniformity of particle sizes within a sample. A low PDI (close to zero) indicates that the particles are nearly the same size, which is often desirable for applications requiring consistent behavior and performance. Conversely, a high PDI suggests a wide range of particle sizes, which can affect the stability and functionality of the nanoemulsion. In this study, the PDI value of 0.224 indicated that, although the nanoemulsion was relatively homogeneous, it was not entirely monodisperse. For comparison, Ghorbanzade et al.^27^ obtained β-cyclodextrin (BCD)-loaded FO with a size of 409 nm and a PDI value of 0.557, and Ojagh and Hasani^28^ reported nanoliposome containing FO with droplet size and PDI values of 339.2 nm and 0.426, respectively. In a study conducted by Li et al.,^29^ it was determined that the size of nanoemulsions containing FO ranged between 245 and 422 nm and PDI between 0.23 and 0.42. These comparisons highlight that the nanoemulsions in this study had a more uniform size distribution.

The zeta potentials of the nanoemulsions were also measured using the same instrument. Zeta potential is a measure of the electrostatic potential at the particle surface and provides information about the stability of the colloidal system. High absolute values of zeta potential (either positive or negative) suggest strong repulsion between the particles, which helps prevent aggregation and thus indicates a stable colloidal system. The zeta potential of the nanoemulsion in this study was found to be −75.33 mV, suggesting a highly stable system due to the strong negative charge, which prevents droplet fusion.^30^ It is believed to be negatively charged due to the fatty acids. Ceylan et al.^31^ reported that the zeta potential of carvacrol-loaded nanoemulsions was −57.24 mV. Similarly, Meral et al.^32^ determined a zeta potential value of −24.8 mV in nanoemulsions containing thyme oil. In the present study, nanoemulsions with an average diameter of 197 nm, a PDI of 0.224, and a zeta potential of −75.33 mV were obtained. Samples exhibiting PDI values of 0.3 or below, such as those observed in the prepared nanoemulsions, indicate that they can be classified as monodisperse, which suggests that the particles are uniformly distributed.^33^ Also, generally, systems with a zeta potential greater than ±30 mV are regarded as stable.^34^ In the study conducted by Zhang et al.,^35^ the zeta potential values were found to be between −37.16 and −50.07 mV, indicating that these values signified stable nanoemulsions. Consequently, in the present study, the nanoemulsion was stable, anionic, and had a uniform distribution.

**Figure 1:**
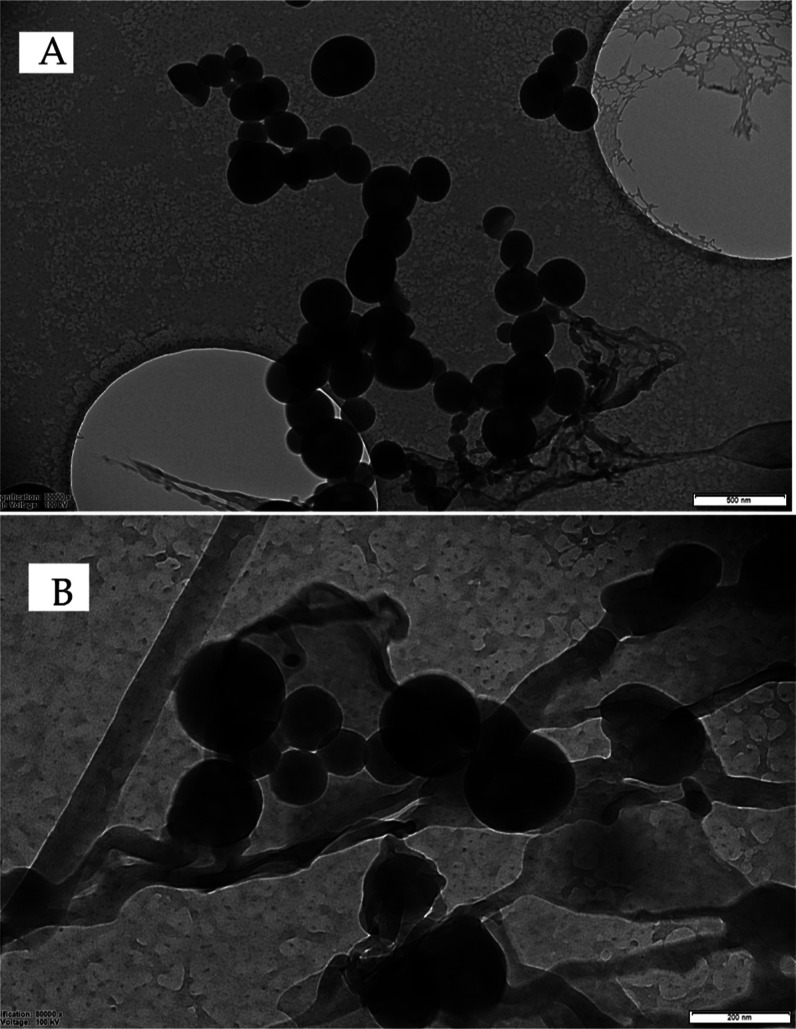
Cryo-TEM images of FO-loaded nanoemulsion.

Figure 1A,B displays the morphological properties of FO-loaded nanoemulsions. Nanoemulsion droplets with spherical smooth surfaces and an average size below 200 nm, which are consistent with DLS data, were obtained.

### 3.2 Proximate Composition

The average nutritional component values for the cookie samples are listed in Table 2. The moisture value of cookies ranged from 6.24 to 7.32%. Fat-free control (FfC) showed the highest moisture content, but there were no statistical differences among samples (*p* > 0.05). The obtained moisture content was within the acceptable moisture range. Dundar^36^ stated that the moisture content of cookies varied between 6.31 and 6.35%. The moisture content described by Yildiz and Gocmen^37^ for gluten-free cookies was between 5.82 and 5.91%. Felisberto et al.^38^ found an average of 5.50% moisture content, in which fat was reduced by 50%. Yalcin^39^ found that biscuits containing ground yellow poppy seed had a moisture level that ranged from 5.85 to 7.19%.

**Table 2:** Nutritional Composition of Cookies^a^

chemical composition	Cont	FoN	FfC
moisture	6.86 ± 0.96^a^	6.24 ± 0.07^a^	7.32 ± 0.04^a^
ash (%)	0.75 ± 0.05^b^	0.90 ± 0.01^a^	0.76 ± 0.02^b^
protein (%)	5.60 ± 0.01^a^	5.46 ± 1.52^a^	6.45 ± 0.06^a^
fat (%)	27.71 ± 0.60^a^	14.18 ± 0.35^b^	14.46 ± 0.79^b^
carbohydrate (%)	59.08 ± 0.29^b^	73.22 ± 1.14^a^	71.02 ± 0.79^a^
energy (kcal/100 g)	508 ± 6.65^a^	442 ± 1.56^b^	440 ± 3.76^b^

a Cont: control cookies, FfC: fat reduced and without containing nanoemulsion cookies, and FoN: cookies containing FO-containing nanoemulsion. Values are given as mean ± standard deviation. Letters a-b indicate groups of samples that are statistically different from each other based on Tukey’s test at the *p* < 0.05 level. Samples sharing the same letter are not significantly different from each other.

Cookies had an ash content of 0.75–0.90%. When compared to the control and fat-reduced samples, FO-loaded samples had a significantly higher (*p* < 0.05) content in total mineral elements (0.90%). Felisberto et al.^38^ reported 0.42–0.63% ash content in the cookies. The protein content of the samples ranged between 5.46% (FoN samples) and 6.45% (Fat-reduced FfC sample). Similar findings were obtained by Felisberto et al.,^38^ who reported that the protein content was 6.98% of the fat-reduced cookie. Significant differences were observed for fat contents among the formulations, with values of about 14% fat content, which were expected, since the fat was reduced at a level of 50%.

Cookies’ energy contents ranged from 440 (fat-reduced FfC) to 508 kcal/100 g (control). Regarding the energy value, a significant reduction in FoN was observed, which presented about 13% fewer calories when compared to the full-fat-containing control sample.

### 3.3 Surface Crack

**Figure 2:**
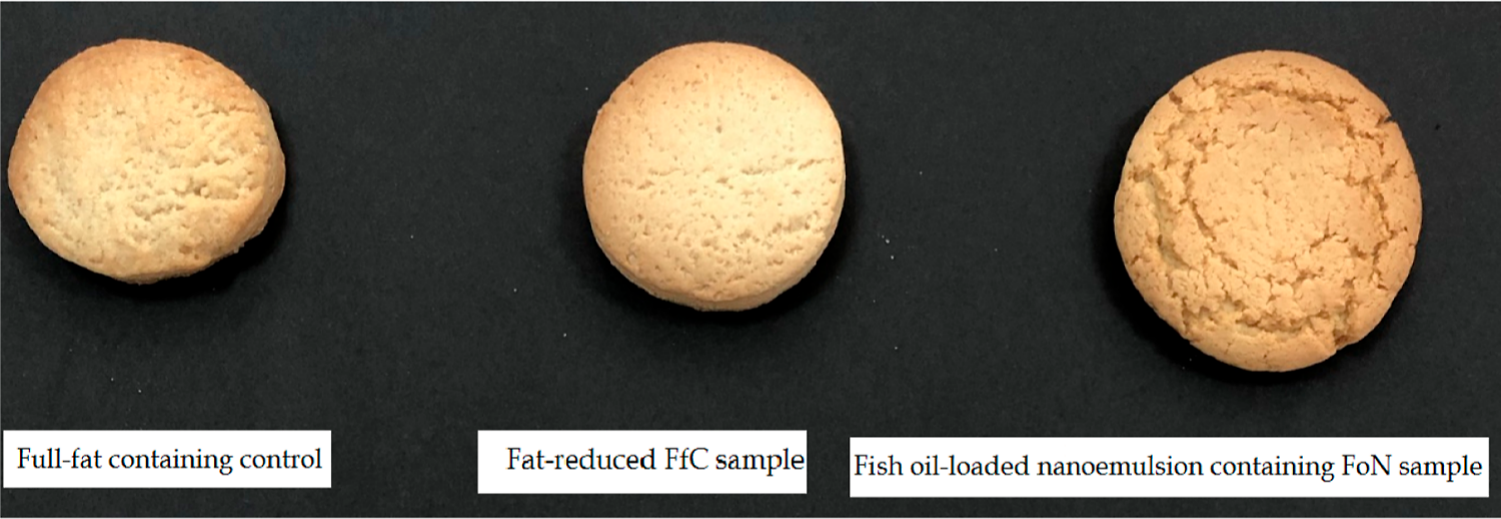
View of cookie samples.

A crucial quality criterion for cookies is their exterior appearance. Figure 2 shows the cookie surface appearance. Nanoemulsion caused homogeneous crack patterns compared with the other two samples. With the addition of a nanoemulsion, the number of islands on the cookie surface increased. Surface cracks were larger, deeper, and more prominent on the FoN cookies. Oven type, ingredients, and fat affect the surface properties. The properties of the surface were also impacted by spreading during baking.^40^ Their size and soft bite are features of cookie quality. Additionally, cookies made with soft wheat flour must have a uniform surface cracking pattern, in addition to a larger spread. In this context, obtaining uniform surface cracks was an important result of this study.

### 3.4 Physical Characteristics of Cookies

The diameter, thickness, and spread ratio values of cookie samples are given in Table 3.

**Table 3:** Physical Properties of Cookies^a^^,^^b^

physical properties	Cont	FoN	FfC
diamater (mm)	46.33 ± 0.95^c^	57.15 ± 0.14^a^	48.53 ± 0.28^b^
thickness (mm)	7.51 ± 0.03^a^	6.45 ± 0.00^c^	7.43 ± 0.00^b^
spread ratio	6.16 ± 0.11^c^	8.86 ± 0.02^a^	6.54 ± 0.04^b^
hardness-0 th day (N)	43.81 ± 0.98^a^	26.98 ± 1.29^b^	40.69 ± 1.20^a^
hardness-45 th day (N)	47.01 ± 2.76^b^	31.86 ± 1.29^c^	59.22 ± 0.26^a^
hardness-90 th day (N)	57.19 ± 0.28^b^	29.28 ± 1.65^c^	62.56 ± 1.76^a^
L^*^	68.74 ± 4.49^b^	67.42 ± 1.33^b^	74.71 ± 2.11^a^
a^*^	3.75 ± 1.83^c^	6.14 ± 0.42^ab^	3.90 ± 2.20^bc^
b^*^	29.83 ± 1.44^b^	33.47 ± 2.24^a^	29.39 ± 2.95^b^

a Cont: control cookies, FfC: fat reduced and without containing nanoemulsion cookies, and FoN: cookies containing FO-containing nanoemulsion. Values are given as mean ± standard deviation. Letters a-b indicate groups of samples that are statistically different from each other based on Tukey’s test at the *p* < 0.05 level. Samples sharing the same letter are not significantly different from each other.

b L*: represents lightness, a*: represents the green to red axis, and b*: represents the blue to yellow axis.

According to the results, the diameter, thickness, and spread ratio values varied between 46.33 and 57.15 mm, 6.45–7.51, and 6.16–8.86, respectively. According to Devi and Kahatkar,^41^ while the diameter of the cookies prepared with various fats and oils ranged from 87.00 to 91.65 mm, cookies had a thickness that varied from 8.30 to 8.60 mm. In the same study, the spread ratio of cookies was determined as 10.29 to 11.04. Chugh et al.^42^ stated that the cookies’ diameter, thickness, and spread ratio ranged from 6.08 to 7.03 cm, 0.57 to 0.73 cm, and 8.44 to 11.96, respectively. In this sense, our results were in accordance with previous studies.

In the present study, replacing fat with a nanoemulsion significantly increased the diameter, but it decreased the thickness values of cookies (*p* < 0.05). A key aspect of cookie quality is cookie diameter. Larger diameter and lesser thickness values were linked to a cookie’s favorable properties.^43^ As can be seen from the results, with nanoemulsion addition, higher-quality cookies with greater diameters were obtained compared to full-fat-containing cookies.

The spread ratio is a measurement of the cookie quality. Cookies with a higher spread ratio are desirable for the consumer.^6^ The amount of fat in the cookie determines its final dimensions. The granules of protein and starch are covered by fat, which isolates them and breaks up the continuity of the structure that protein and starch create.^44^ When fat found in the dough formulation melts during baking, the cookie dough expands. In this context, fat contributes to the expansion of dough. But, when fat is reduced, the expansion of dough is limited. Decreased cookie spread results from reducing the fat level of cookie formulations, which has a detrimental influence on the final cookie quality.^45^ Filipčev et al.^46^ found a significant linear relationship (*R*^2^ = 0.92) between the cookie spread ratio and fat content in the formulation. In the studies of Sudha et al.^47^ and Pareyt et al.,^44^ a larger spread ratio of cookies with a higher fat content was also found.

In a study conducted by Erinc et al.^48^ when the formulation’s fat content was reduced by 75%, the spread ratios for biscuits containing 40% shortening reduced from 6.1 to 4.3. They stated that the cookies with lower spread ratio values and the thicker ones were obtained with increasing levels of fat reduction. Similar to this, Laguna et al.^49^ and Zbikowska et al.^50^ observed that substituting fat mimetics for actual fat had an impact on biscuit shape by increasing thickness and reducing the spread ratio.

In the present study, the spread ratio of nanoemulsion-containing cookies increased compared with full-fat-containing cookies and fat-reduced cookies (FfC). The value was 8.86 for the nanoemulsion-containing cookie. Although previous studies identified a reduction in diameter and spread values with fat reduction, our study achieved higher diameter and spread ratio values compared with the full-fat-containing control sample. The result was attributed to the expansion of the surface area of the oil, whose size was reduced to the nanoscale, and covered the entire surface of the dough.^12,17,51^ In summary, a little amount of nanosized oil fulfilled the function which is achieved by a larger amount of its bulk counterparts.

### 3.5 Hardness

The hardness is associated with a human bite and is related to the applied force that produces sample rupture or deformation.^52^ The effect of fat reduction on the hardness of cookies is shown in Table 3. On the initial storage day, the nanoemulsion addition decreased the hardness of the cookie significantly compared to the full-fat-containing cookie (control) and fat-reduced cookie group (FfC). The control sample had the maximum hardness value (43.81 N), whereas the nanoemulsion-containing group had the lowest hardness value (26.98 N).

During the 90 day storage period, nanoemulsion-containing cookies with a 29.28 N hardness value were softer than full-fat-containing cookies (57.19 N) and fat-reduced cookies (62.56 N). With nanoemulsion addition, the hardness of cookies first increased on the 45th day of storage and, then on the 90th day of storage, nanoemulsion addition of up to 50% decreased the hardness of the cookies. During storage, the hardness of cookies increased in the full-fat-containing control and fat-reduced samples but remained stable in the FO-loaded nanoemulsion-containing samples. The hardest sample was the fat-reduced cookie (FfN), with a final hardness value of 62.56 N.

The previous findings suggested that fats had a substantial impact on the textural characteristics of cookie dough.^41^ When a portion of the fat was removed, the texture became the main problem: the cookie became crumbly and^48^ harder. However, in the present study, a decrease in the hardness value was observed in the nanoemulsion-containing cookies.

Xie et al.^53^ stated that when the fat replacement rate was more than 35%, the hardness of low-fat cookies was increased significantly (*p* < 0.05)*.* Similarly, Erinc et al.^48^ found that the use of some fat mimics (various particle sizes and different amounts of plant fibers) increased the hardness of low-fat biscuits.

Depending on the fat and flour characteristics in the cookie recipe, the textural characteristics can be changed. The use of shortening and soft wheat flour in the baking process ensures that the cookies had the desired crunch. One of the key elements influencing the quality and textural characteristics is crunchiness.^37^ In biscuits, the fat covers the protein to create a crumbly structure and helps incorporate air for a softer texture.^54^ In the present study, the decrease in hardness of nanoemulsion-containing cookies as compared to control cookies was due to the cover of gluten protein with nanosized oil. Probably, nanosized oil covered the whole surface of the flour, resulting in a softer and crunchier texture. It is important to note that when discussing the impact of the nanoemulsion, we refer to the combined effect of the oil, water, and surfactant mixture. The surfactant, in particular, plays a crucial role in influencing the texture, appearance, and other properties of the cookies.

### 3.6 Color

One of the most crucial considerations when choosing food goods is color. Table 3 illustrates the impact of replacing fat with a nanoemulsion on the color parameters of cookies. L* represents lightness and ranges from 0 (black) to 100 (white), indicating how light or dark the color is. a* represents the green to red axis, with positive values indicating red and negative values indicating green. b* represents the blue to yellow axis, with positive values indicating yellow and negative values indicating blue. The cookies’ L* values ranged from 67.42 to 74.71. The L* value of the FfC sample is the highest value. The L* values of the full-fat-containing sample and nanoemulsion-containing sample did not affect statistically (*p* > 0.05). According to Dundar,^36^ the L* value of the control cookie was 74.18. Yalcin^39^ observed the decrease (from 71.12 to 67.49) in L* value with fat reduction, and the author attributed the results to that low-fat content could serve as a plasticizer to cover all powder ingredients that created a Maillard reaction with a lot of sugar.

Browning is the term used to describe the coloration that develops during baking of bakery products. The Maillard reaction and caramelization are two examples of nonenzymatic chemical processes that result in colored chemicals during baking and cause browning.^55^ In this sense, the L* value of cookies came from the Maillard reaction, caramelization, and the nature of used ingredients. Also, Ojagh and Hasani^28^ showed that the lightness of bread was decreased due to the FO microcapsule content increasing.

When the color of the cookies was examined, a* and b* values of the cookies ranged between 3.75 and 6.14 and 29.39 and 33.47, respectively (Table 3). The b* value was significantly increased in the nanoemulsion-containing cookie, indicating a greater influence of yellowness due to the presence of FO. Ojagh and Hasani^28^ found that the FO release was the cause of the rise in b* values of bread crumbs.

L* value was decreased as a result of the nanoemulsion, which also increased the a* and b* values. Similar results were also observed by Yalcin^39^ (2017), who reported a decrease in the L* value and an increase in the a* and b* values of cookies. As a result, FO-loaded nanoemulsions had a notable influence on cookie color during baking.

### 3.7 Sensory Properties

**Figure 3:**
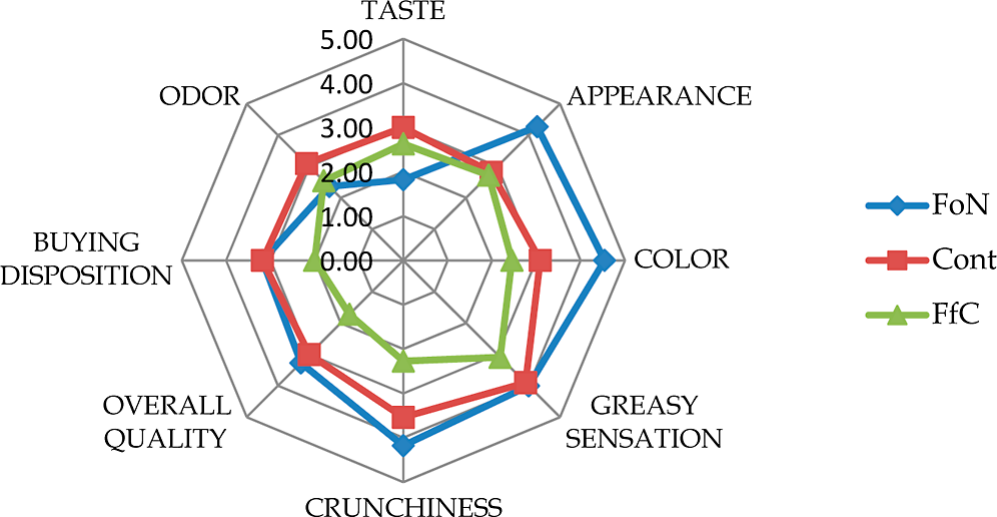
Sensory attributes of cookie samples.

To determine whether consumers would prefer the cookies or not, a sensory panel was performed. Figure 3 shows the results of the evaluation of appearance, taste, odor, greasy sensation, color, crunchiness, overall quality, and buying intention. The majority of panelists liked the appearance of FO-loaded-containing cookies regardless of the formulation. Curiously, the fat reduction did not affect the ″greasy″ sensation, which is an important finding since it suggests that fat content might be reduced by 50% without detracting from the desirable greasy sensation and yet have a positive impact on health The greasy sensation refers to the sensory perception of smoothness and slipperiness that is typically associated with the presence of fat in food products. This sensation contributes significantly to the mouthfeel and overall sensory experience of bakery products. The perception of greasy is influenced by the amount and type of fat used in the formulation.^56,57^ The use of FO-loaded nanoemulsions not only helped in maintaining the greasy sensation but also enhanced the nutritional profile of the cookies by increasing the content of polyunsaturated fatty acids (PUFA) and omega-3 fatty acids, specifically EPA and DHA. Thus, the incorporation of nanoemulsions allowed for a reduction in overall fat content while still providing the sensory benefits associated with higher fat levels. The results from the sensory panel indicated that the nanoemulsion-containing cookies received high scores for greasy sensation, comparable to that of full-fat cookies. This suggested that the nanoemulsion technology successfully replicated the mouthfeel of traditional fats, making it a viable option for reducing the fat content in baked goods while maintaining consumer acceptance. In conclusion, the incorporation of FO-loaded nanoemulsions in cookie formulations provided a means to reduce fat content by up to 50% without compromising the desirable greasy sensation. This approach not only supported the development of healthier baked goods but also enhanced their nutritional value, aligning with the growing consumer demand for health-conscious food options.

FO-loaded-containing cookie was perceived better than the control sample in terms of color and appearance attributes. When asked if they would purchase the cookies, the panelists indicated that they would be least likely to buy the fat-reduced cookie. On the contrary, they turned out to be more willing to buy cookie samples containing nanoemulsions.

Regarding the color, appearance, greasy sensation, and crunchiness scores, nanoemulsion-containing cookie samples gained 4.54, 4.27, 4, and 4.18 scores, respectively, which showed consumers’ acceptance of the tested cookie samples as “like slightly″. On other the hand, the control samples gained lower scores compared to FoN cookies for these properties.

The cookies in this study had an overall acceptability score that varied from 2 (a fat-reduced cookie) to 3.27 (a nanoemulsion-containing cookie).

When the samples’ tastes were assessed, it was found that cookies containing FO-loaded nanoemulsion were the least well-liked, whereas control cookies were the most well-liked. While the addition of FO-loaded nanoemulsion had no negative effects on practically all sensory attributes, taste scores decreased with the addition of FO-loaded nanoemulsion. The undesirable taste of FO was also felt in the cookies. Previous studies^20,58^ reported that the undesirable taste of FO was felt in the food products enriched with FO, and a taste-masking agent could be added to the formula. In a study conducted by Ghorbanzade et al.,^17^ the panelists gave the control yogurt sample the highest score for taste and aroma as well as overall acceptance. This was mostly due to the distinct flavor of FO in yogurt as compared to that in the control sample.

The crunchiness scores of the FoN sample were higher than those of the control cookies. Gluten plays an effective role in the formation of cookie crunchiness, which means better quality when crunchiness is high. The fat, called shortening, competes with water for the surface of the flour, and gluten formation is limited as the water required for gluten formation is reduced and thus crunchiness occurs. Due to a greater development of the gluten network, fat-reduced cookies exhibit higher hardness and brittleness and poorer crumbliness than their full-fat counterparts.^47,49,59,60^ Therefore, the fat reduction can cause harder texture, and crunchiness is reduced. In the present study, the mentioned problems did not occur, and even softer and more crispy cookies were obtained compared to full-fat biscuits. The success was obtained with only 2 g nanosized oil, which indicated that nanomaterials having larger surface areas have many advantages compared to their bulk counterparts. As a result, the sensory score of FO-loaded nanoemulsion-containing cookies was found to be equal (*p* > 0.05) or higher to the control cookies, except for taste scores.

### 3.8 Fatty Acid and Lipid Quality Indexes of Cookies

The findings of the examination of the fatty acid composition of the cookie samples are shown in Table 4. While a total of 19 fatty acids in nanoemulsion-loaded samples were identified, the number was 17 for control and fat-reduced control samples, in which C22:5n3 and C22:6n3 (docosahexaenoic acid: DHA) were not present in these samples. The major fat constituents in all cookies included palmitic (C16:0; 46.18–42.90%), oleic acid (C18:1; 30.67–33.61%), and linoleic acid (C18:2n6; 10.94–11.93%). Dias et al.^61^ stated that 35.31–50.64% palmitic acid, 31.43–48.94% oleic acid, and 10.87–20.34% linoleic acid were found in salty biscuits. In this sense, our findings are consistent with the findings of Dias et al.^61^ The large portions of palmitic and oleic acids came from shortening. About 42% palmitic acid and 35% oleic acid were determined in emulsified margarine by Culetu et al.^44^ In all samples, these fatty acids were found, but there were no statistical differences between cookies in terms of palmitic (C16:0), stearic (C18:0), oleic (C18:1), and linoleic acid (C18:2) contents. On the other hand, the FoN cookie was the only cookie that contained C22:5n3 and C22:6n3 (docosahexaenoic acid: DHA). As compared to other cookies, FO-loaded nanoemulsion-containing cookies were found to be the cookies containing the highest percentage of polyunsaturated fatty acids (PUFA). The amount of C20:5n3, called eicosapentaenoic acid (EPA) and known to help cardiovascular health and the formation of nerve and brain cells,^63^ was 5 times higher than the full-fat-containing control cookies.^62^

**Table 4:** Fatty Acid Composition and Lipid Quality Indexes^a^

fatty acid	Cont	FoN	FfC
C8:0	0.04 ± 0.00^a^	0.055 ± 0.014^a^	0.07 ± 0.002^a^
C10:0	0.034 ± 0.002^a^	0.043 ± 0.010^a^	0.047 ± 0.005^a^
C12:0	0.23 ± 0.006^a^	0.25 ± 0.0040^a^	0.024 ± 0.012^a^
C14:0	0.96 ± 0.004^b^	1.46 ± 0.015^a^	0.98 ± 0.002^b^
C14:1	0.048 ± 0.000^a^	0.055 ± 0.038^a^	0.052 ± 0.002^a^
C16:0	46.18 ± 0.009^a^	42.90 ± 0.26^a^	43.72 ± 2.04^a^
C16:1	0.26 ± 0.009^b^	0.56 ± 0.001^a^	0.27 ± 0.030^b^
C17:0	0.15 ± 0.08^a^	0.28 ± 0.04^a^	0.28 ± 0.095^a^
C17:1	0.041 ± 0.006^a^	0.096 ± 0.005^a^	0.038 ± 0.05^a^
C18:0	7.87 ± 0.029^a^	7.45 ± 0.10^a^	7.73 ± 0.309^a^
C18:1n9	31.91 ± 0.34^a^	30.67 ± 0.38^a^	33.61 ± 1.58^a^
C18:2n6c	10.94 ± 0.25^a^	10.97 ± 0.13^a^	11.93 ± 0.67^a^
C18:3n6	0.38 ± 0.008^a^	0.38 ± 0.01^a^	0.40 ± 0.05^a^
C18:3n3	0.11 ± 0.004^a^	0.81 ± 0.68^a^	0.31 ± 0.00^a^
C20:0	0.18 ± 0.07^a^	0.20 ± 0.05^a^	0.19 ± 0.05^a^
C20:4n6	0.29 ± 0.006^b^	0.70 ± 0.04^a^	0.00 ± 0.00^c^
C20:5n3-EPA	0.34 ± 0.09^b^	1.85 ± 0.04^a^	0.13 ± 0.18^b^
C22:5n3	0.00 ± 0.000^b^	0.46 ± 0.08^a^	0.00 ± 0.00^b^
C22:6n3-DHA	0.00 ± 0.000^b^	0.77 ± 0.007^a^	0.00 ± 0.00^b^
ΣSFA	55.66 ± 0.011^a^	52.66 ± 0.05^a^	53.26 ± 2.52^a^
ΣMUFA	32.26 ± 0.24^a^	31.38 ± 0.41^a^	33.98 ± 1.67^a^
ΣPUFA	12.07 ± 0.25^b^	15.95 ± 0.36^a^	12.76 ± 0.85^b^
Σn-3	0.46 ± 0.01^b^	3.90 ± 0.55^a^	0.43 ± 0.18^b^
Σn-6	11.60 ± 0.26^a^	12.05 ± 0.18^a^	12.32 ± 0.66^a^
n-3/n-6	0.04 ± 0.00^b^	0.32 ± 0.05^a^	0.03 ± 0.01^b^
PUFA/SFA	0.22 ± 0.00^b^	0.30 ± 0.00^a^	0.24 ± 0.02^b^
AI	1.30 ± 0.00^a^	1.18 ± 0.00^a^	1.19 ± 0.11^a^
TI	1.55 ± 0.01^a^	1.04 ± 0.03^b^	1.41 ± 0.16^a^
HH	0.92 ± 0.00^a^	1.04 ± 0.00^a^	1.03 ± 0.10^a^

a Cont: control cookies, FfC: fat reduced and without containing nanoemulsion cookies, and FoN: cookies containing FO-containing nanoemulsion. Values are given as mean ± standard deviation. Letters a-b indicate groups of samples that are statistically different from each other based on Tukey’s test at the *p* < 0.05 level. Samples sharing the same letter are not significantly different from each other. SFA: saturated fatty acids, EPA: eicosapentaenoic acid, DHA: docosahexaenoic acid, AI: atherogenic index, TI: thrombogenic index, HH: hypocholesterolemic/hypercholesterolemic ratio, MUFA: monounsaturated fatty acids, and PUFA: polyunsaturated fatty acids. Fatty acid ratio is given as %.

The total n-3 fatty acid content of cookies increased from 0.46% (in control cookies) to 3.90% (in nanoemulsion-containing cookies). The n3/n6 ratio varied between 0.03 for FfC samples, 0.04 for the control, and 0.32 for FoN samples. The n-3/n-6 ratio is a useful metric for assessing the relative nutritional value of an oil. To reduce the risks of cancer, excessive plasma cholesterol levels, and coronary heart disease, a larger ratio is crucial. The n-3/n-6 ratio of samples containing nanoemulsions was about 10 times higher than control cookies. As shown in Table 4, the cookies with nanoemulsion had the highest ratio of PUFA/SFA (0.30), whereas the control cookies had the lowest (0.22). A higher PUFA/SFA ratio is significantly healthier.^64^ Of course, since these fatty acids are found in FO, the results were also expected to be found in cookies containing FO. However, with a very low amount of FO (2 g), an increase in the quality characteristics of the cookies compared to the control and the presence of these fatty acids was also an important success.

The AI and TI were investigated in this study to evaluate the potential health benefits of FO-loaded nanoemulsion cookies. These indices are important markers for assessing the impact of dietary fats on cardiovascular health. The AI is a measure used to evaluate the risk of developing atherosclerosis, a condition characterized by the buildup of fatty deposits in the arteries that can lead to cardiovascular diseases such as heart attacks and strokes. The AI is calculated based on the ratio of certain fatty acids that are known to influence cholesterol metabolism. A lower AI indicates a lower risk of atherosclerosis and is therefore considered to be more beneficial for cardiovascular health. The TI is another measure used to assess the potential risk of thrombosis, which is the formation of blood clots that can obstruct blood vessels and lead to conditions such as heart attacks and strokes. The TI takes into account the balance between prothrombogenic (clot-promoting) and antithrombogenic (clot-preventing) fatty acids. A lower TI indicates a lower risk of thrombosis and is thus more favorable for cardiovascular health.^65−68^ In this context, evaluating the AI and TI indices helped in understanding the broader implications of substituting traditional fats with FO-loaded nanoemulsions in cookie formulations. The results of our study, as shown in Table 4, provide a clear comparison of these indices across different sample groups. In the present study, the control group had a higher AI (1.30) compared to the FfC and nanoemulsion-loaded groups (1.19 and 1.18, respectively). However, there was no statistical difference among the samples. The TI score of the FoN group (1.04) was lower than that of the control (1.55) and FfC group (1.41) (*p* < 0.05).

A higher HH is desired.^69^ Although there was no statistical difference among cookies, the HH index of the samples containing the nanoemulsion was higher than the other samples.

From a nutritional point of view, nanoemulsion-containing cookies showed the significantly (*p* < 0.05) highest values of C20:4n6, EPA, DHA, PUFA, total n-3 fatty acid, n-3/n-6, TI, and PUFA/SFA. Finally, AI, TI, and H/H, strictly related to the fatty acid profile, showed the best values in nanoemulsion-containing cookies.

## 4 Conclusions

In this study, a 2 g nanosized FO-loaded nanoemulsion was utilized as a fat replacer in cookie formulations. The effects of this fat replacement on various attributes of the cookies, including diameter, thickness, spread ratio, hardness, color values, fatty acid profiles, and sensory properties, were thoroughly investigated. This study successfully demonstrated the potential of FO-loaded nanoemulsions as effective fat replacers in cookie formulations, offering significant health benefits without compromising sensory qualities. The use of nanoemulsions enabled the reduction of overall fat content while maintaining a desirable greasy sensation and enhancing the nutritional profile with higher levels of beneficial omega-3 fatty acids (EPA and DHA).

The incorporation of FO-loaded nanoemulsions resulted in cookies with improved cardiovascular health markers, as indicated by lower AI and TI. This innovative approach aligns with the growing consumer demand for healthier food products and demonstrates the feasibility of using nanotechnology to create functional foods with enhanced health benefits.

Overall, the findings suggest that FO-loaded nanoemulsions can be an effective strategy for producing low-fat baked goods that do not sacrifice taste or texture. This study provides valuable insights for the food industry, paving the way for the development of a variety of health-conscious bakery products that cater to the nutritional needs of consumers; future research should focus on optimizing the formulation to further mask any residual FO taste and exploring the application of nanoemulsions in other food products. The promising results from this study highlight the potential of nanoemulsion technology in advancing food science and improving public health through better nutrition.
